# Relation between the strength and dimensionality of defect-free carbon crystals

**DOI:** 10.1186/s11671-015-0929-9

**Published:** 2015-05-19

**Authors:** Sergiy Kotrechko, Andrey Timoshevskii, Eugene Kolyvoshko, Yuriy Matviychuk

**Affiliations:** G.V. Kurdyumov Institute for Metal Physics, Kyiv, Ukraine; Taras Shevchenko Kyiv National University, Kyiv, Ukraine

**Keywords:** Dimensionality, Strength, Coordination number, Diamond, Graphene, Carbyne

## Abstract

On the basis of ab initio simulations, the value of strength of interatomic bonds in one-, two- and three-dimensional carbon crystals is obtained. It is shown that decreasing in dimensionality of crystal gives rise to nearly linear increase in strength of atomic bonds. It is ascertained that growth of strength of the crystal with a decrease in its dimensionality is due to both a reduction in coordination number of atom and increase in the angle between the directions of atomic bonds. Based on these data, it is substantiated that the one-dimensional (1D) crystals have maximum strength, and strength of carbyne is the absolute upper limit of strength of materials.

## Background

According to the existing paradigm, there are two ways to increase strength, namely (i) by increase in the density of defects in crystal lattice (increase in a lattice distortion) or (ii) vice versa, creation of defect-free crystals. The first way to increase strength is the basis of material science of conventional “bulk” materials. Different kinds of mechanical and thermo-mechanical treatments of metals and alloys are, in most cases, aimed at increasing the distortions in the matrix, which increases the resistance to movement of dislocations, i.e., increases the strength of the metals or alloys. Removal of defects is an alternative way to increase the material strength. Nanosized crystals are an example of this [1–3]. However, new factor appears in this case, which influences strength essentially. This is dimensionality [4–6]. Most clearly, this effect can be demonstrated for allotropic forms of carbon. Thus, the strength of defect-free 3D crystal (diamond) is 95 GPa [7], of 2D-crystal (graphene)—130 GPa, [8] and of 1D crystal (carbyne)—more than 270 GPa [9]. These numbers are evidences for the key role of dimensionality in “governing” strength in a nanoworld. In this connection, the task of the work was to establish the regularities of influence of the crystal dimensionality on its strength and to develop ideas about the factors influencing this effect.

## Methods

One-, two-, and three-dimensional carbon crystals were used as an object of investigation. To determine the strength of a one-dimensional crystal (monatomic chains of carbon atoms), ab initio calculations were employed. In this case, tension of the chain was simulated. Strength was determined for polyyne structure, which is energy-wise more favorable. In this paper, the pseudo-potential method was employed, which is successfully used for all calculations of the atomic and electronic structures of crystals and which is based on the translational symmetry. When modeling the atomic structure of infinite chains of atoms by this method, it is necessary to eliminate their interaction with each other. The model ordered structure with unit cell parameters *a* = *b* = 1 nm and *с* = 0.2566 nm was used to this end. Tension of chains was executed by changing parameter *с*. Total energies of chains were calculated by the pseudo-potential method (software package Quantum-ESPRESSO (QE)) [10]. This method was used for modeling the mechanical properties of the chains. Pseudo-potentials for carbon were generated under the scheme Vanderbilt Ultrasoft with the package Vanderbilt code, version 7.3.4 [11]. The exchange-correlation potential PBE [12] was used. For the calculations, we use 51 *k* points in irreducible Brillouin zone. The value of the cutoff energy *E*cu_t_ = 450 eV. Structural optimization of positions of the atoms in the chain in longitudinal and transverse directions was carried out within the accuracy 1 mRy/a.e. The accuracy of calculation of total energies was 0.001 eV.

To determine the strength of atomic bonds, *R*_2D_, in two-dimensional (2D) crystal (graphene), the value of fracture stress of graphene [13] obtained by ab initio simulation of tension of graphene sheets in two directions “zigzag” and “armchair” were used. The magnitude of atomic bond strength *F*c was determined as the value of critical force acting on the bond at the time of instability of the graphene sheet at its tension:

1 $$ {R}_{2\mathrm{D}}^{\mathrm{zig}}\kern0.5em =\kern0.5em {\sigma}_{\mathrm{c}}^{\mathrm{zig}}{d}_0{b}_0\kern0.5em \times \kern0.5em \left(1\kern0.5em -\kern0.5em \varepsilon \right) $$

2 $$ {R}_{2\mathrm{D}}^{\mathrm{arm}}\kern0.5em =\kern0.5em {\sigma}_{\mathrm{c}}^{\mathrm{arm}}{d}_0{b}_0\kern0.5em \times \kern0.5em \left(1\kern0.5em -\kern0.5em \varepsilon \right)\times \left(1\kern0.5em +\kern0.5em  \cos \left({\theta}_0\right)\right)\kern0.5em \times \kern0.5em  \cos \left(1\kern0.5em -\kern0.5em {\theta}^{\hbox{'}}\right) $$

where ${\sigma}_{\mathrm{c}}^{\mathrm{zig}}$ and ${\sigma}_{\mathrm{c}}^{\mathrm{arm}}$ are the critical stresses of instability of graphene sheet under tension in the directions zigzag and armchair, respectively; ${b}_0\kern0.5em =\kern0.5em \sqrt{3}/2{a}_0$; *a*_0_ = 0.1422 nm is the lattice parameter; *d*_0_ = 0.334 nm is the effective thickness of graphene sheet; *θ*_0_ = 120° is the angle between atomic bonds ij and jk in undeformed state; *θ*′ = 130° is the angle, at which instability in graphene occurs (was determined based on the geometry of the system at instability); and *ε* is the transverse deformation of graphene sheet.

Strength of atomic bonds in three-dimensional (3D) crystal (diamond) was determined on the basis of ab initio simulations of tension of diamond crystals in the direction <111 > [7]. The strength value was calculated as the critical magnitude of the force acting on the interatomic bond at instability of the crystal under tension:

3 $$ {R}_{3\mathrm{D}}\kern0.5em =\kern0.5em \frac{3\sqrt{3}}{2}{\sigma}_c{a}_0^2{ \cos}^2\left(\pi /2\kern0.5em -\kern0.5em \theta \right) $$

where σ_c_ is the critical stress of instability, *a*_0_ is the lattice parameter, and *θ* is the angle between atomic bonds ij and jk in deformed state.

**Fig. 1:**
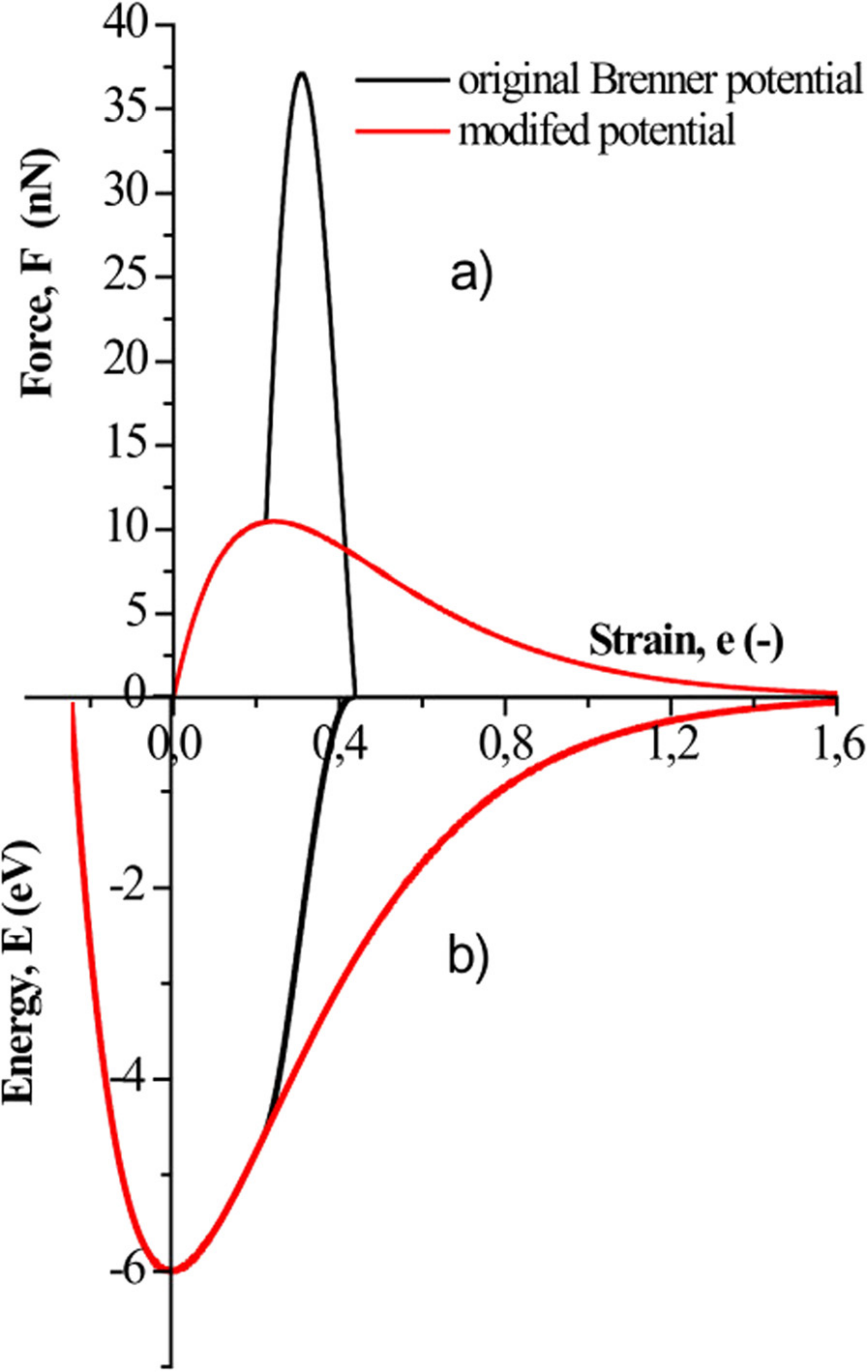
The original and modified Brenner potential. Force vs. strain (**а**). Energy vs. strain (**b**)

Theoretical analysis of the orientation-dependence of strength and its value-dependence on coordination number of atom was executed within the formalism of Brenner potential [14], which is based on Abel pseudo-potential theory [15] that describes well the carbon compounds. Parameterization of the potential was carried out by the set II of Brenner parameters [14]: *R* = 0.139 nm, *D*(e) = 6.0, *s* = 1.22, *β* = 0.21 nm^−1^, *δ* = 0.5, *a*_0_ = 0.00020813, *c*_0_^2^ = 330^2^, and *d*_0_^2^ = 3.5^2^. Since the Brenner potential in initial state gives a significant overestimation of strength of atomic bonds, which reaches values of 30 nN (Fig. 1a), its modification was carried out for the crystal that consisted in correction of cutoff function fcut (rij) ≡ 1. It enabled to get the values of strength, which agree well with the results of ab initio simulations.

## Results and discussion

**Fig. 2:**
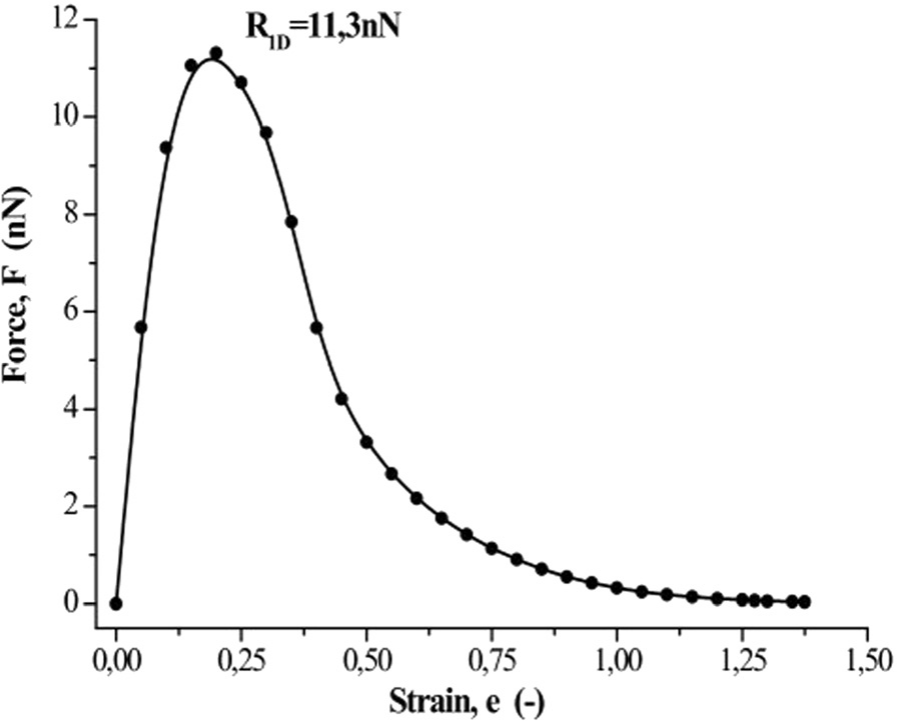
Dependence force vs. strain for infinite monatomic chain. Result of ab initio calculations

The strength of atomic bonds in one-dimensional crystal was determined by the critical stress of instability of monatomic chain under the conditions of uniaxial tension (Fig. 2). In accordance with the data obtained, *R*_1D_ = 11.3 nN. This value is in good agreement with the evidence of 12.2 nN [16] and with the value 12.3 nN obtained for chains containing more than ten atoms [4].

**Fig. 3:**
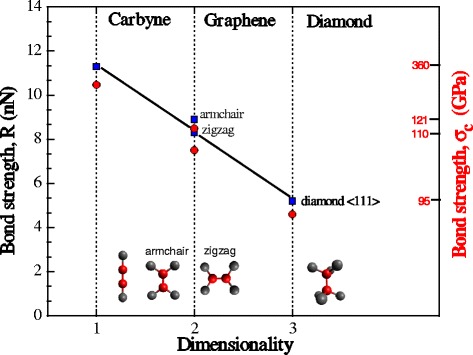
Dependence of the strength of interatomic bonds of carbon crystals on the its dimensionality: *blue squares* are the strength values obtained by the results of ab initio calculations and *red circles* are the results of calculation by the modified Brenner potential for angles between interatomic bonds in the unstrained state and at the critical strain of crystal instability, respectively

To determine the strength of atomic bonds in 2D-crystals, data of ab initio simulation of tension and fracture of graphene sheets in zigzag and armchair direction [13] were used. The critical value of the force at the moment of instability of the graphene sheet was determined by Eqs. (1) and (2). According to the results obtained, the strength of atomic bonds in graphene for armchair direction was 8.9 nN and for zigzag direction—8.3 nN. It should be noted that the difference in angles between atomic bonds in the stretched sheet of graphene is the cause for different values of atomic bonds strength in graphene at transition from zigzag to armchair orientation. Thus, at the moment of instability under tension in armchair direction, the orientation of atomic bonds is characterized by angles of 130° and 130°, while for tension in zigzag direction, they are 114° and 132°, respectively (Fig. 3). Really, calculations using Brenner potential, in the first case, give the value of strength of atomic bonds equal to 8.49 nN, and in the second case, this value is 7.5 nN (Fig. 3). Accordingly, the ratio of the strengths of atomic bonds obtained by the result of ab initio calculations is 1.07 and of those obtained from the Brenner potential is 1.14. The difference does not exceed 9 %. The absolute value of these “strength anisotropy” is not much great, but it illustrates one of the specific features of interatomic interaction of carbon atoms, namely, its orientation-dependence.

The value of bond strength in diamond was obtained based on the results of ab initio simulations. The critical value of the force acting in the bonding at the time of instability of the diamond lattice was determined by Eq. (3). In compliance with the results obtained, bond strength in diamond is *R*_*3D*_ = 5.2 nN.

According to the results, change in the dimensionality of defect-free crystals essentially affects their bond strength. This effect manifests itself in a significant (more than two times) increase in the bond strength of the transition from three- to two- to one-dimensional crystals (Fig. 3). Strength values of bonds calculated using the modified Brenner potential are plotted on the same graph. Calculations were executed for both the initial crystal and taking into account changes of angles *θ* between the bonds when reaching a critical strain of crystal instability. The results of calculations, agree well with the ab initio data. As it is indicated in Fig. 3, the transition from three- to two- to one-dimensional crystals, change in coordination numbers is accompanied by a change in the angles *θ* between the directions of atomic bonds. To separate the contributions of the coordination number *Z* and the angle *θ* to this change in the strength *R*, Brenner formalism was used. In explicit form, the dependence of strength *R* on the coordination number *Z* may be derived only for the case of equal angles *θ*_ijk_ with all neighboring atoms *k*:

4 $$ R\left(Z,\theta \right)\kern0.5em =\kern0.5em {D}^{(e)}\beta \frac{\sqrt{2s}}{s\kern0.5em -\kern0.5em 1}\left({s}^{1/\left(1\kern0.5em -\kern0.5em s\right)}\kern0.5em -\kern0.5em {s}^{s/\left(1\kern0.5em -\kern0.5em s\right)}\right)\kern0.5em \times \kern0.5em {\left(1\kern0.5em +\kern0.5em \left(z\kern0.5em -\kern0.5em 1\right)G\left({\theta}_{\mathrm{ijk}}\right)\right)}^{\frac{\delta s}{1\kern0.5em -\kern0.5em s}} $$

where *G (θ*_ijk_*)* is the angle function [14].

**Fig. 4:**
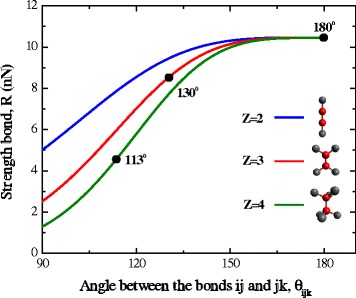
Influence of bonds orientation on the strength of carbon crystals at a fixed value of coordination number *Z. Filled circles*—angles between the bonds at the moment of instability of carbyne (180°), graphene (130°), and diamond (113°)

Using the relations suggested in [14] and the magnitudes of Brenner parameters (set II from [14]), orientation dependencies of strength at fixed values of coordination number *Z* = 2, 3, 4 were built (Fig. 4).

Formally, Brenner-dependence describes the change in strength with the angle within the range from 0° to 180°. But in the real objects (diamond, graphene, carbyne), variation of the angle does not exceed the range 90°–180°, so the analysis is confined to this range of values.

**Fig. 5:**
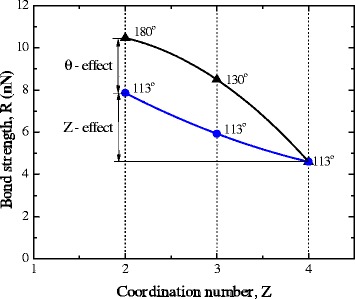
Dependence of the atomic bond strength on the coordination number at the fixed values of angles: *filled triangles* are the critical value of angles; *blue circles* are the atomic bond strength at the fixate values of angles (113°)

According to the data in Fig. 4, the sensitivity of strength to change in the angle increases with the growth of coordination number *Z*. On the other hand, the sensitivity of strength to changes in coordination number increases with decreasing in angle. As it is indicated in Fig. 5, at transition from diamond to carbyne, bond strength increases 2.3 times, while 56 % of this increase in the strength is due to a decrease of the coordination number and 44 % is related to the growth of angle to the maximum possible one, which is 180°.

Regularities of dependence of atomic bond strength on coordination number are demonstrated by the carbon, because it is most convenient to analyze this problem. However, these regularities are inherent also to other materials. Work [17] gives evaluation of the strength of atomic bonds in 1D and 3D crystals for metals such as Cu, Ag, Au, Pd, and Pt. The results of calculations by different methods show that at transition from 1D- to 3D-crystals, the strength of atomic bonds should increase by two to three times.

Such a significant increase in the strength of the interatomic bond is due to the change in electronic structure of the crystal, which occurs with a decrease in the coordination number. In carbon, this manifests itself in the transition from sp^3^- to sp^2^- and sp^1^-hybridization. In metallic crystals, a significant change in the electronic structure is also observed. Qualitatively, this change can be interpreted as an increase in the portion of “covalent” component [18].

Thus, the material in the form of a one-dimensional crystal (monatomic chain) should have the highest level of strength. In this regard, strength of carbyne claims to be the absolute upper limit of strength of materials. According to the results of ab initio calculations, carbyne containing five atoms has a maximum strength. This strength is 13.1 nN [4]. When the effective diameter of the chain equals 0.200 nm [9], its value is 417 GPa. In [19], for an infinite chain, the strength values 9.3–11.7 nN were obtained, and considerably smaller value of effective diameter *d* = 0.077 nm was utilized. This gives the strength value for infinite chain equal to 1988–2501 GPa. In [9], the experimental measurement of carbyne strength was executed. It was ascertained that its strength must exceed 270 GPa. This lower bound for strength of carbyne is more than two times greater than the experimental value of strength of graphene (two-dimensional crystal of carbon).

## Conclusions

Increase in strength of the interatomic bonds while reducing in dimension of the crystal is a specific feature of nanosized defect-free crystals. At transition from three- to two- to one-dimensional crystal of carbon, interatomic bond strength increases almost linearly. The bond strength in carbon monatomic chain is 2.2 times higher than that in diamond. This value is of the same order as the increase in strength of metallic crystals, which may be two to three times.

Growth of crystal strength with decreasing in its dimensionality is due to two factors, namely, a decrease in coordination number and increase in the angle between the bonds. At transition from diamond to carbyne, 56 % of increase in strength is due to a decrease in coordination number and 44 %—due to increasing in angle between the bonds.
